# Corrosion behavior of HRB400 and HRB400M steel bars in concrete under DC interference

**DOI:** 10.1038/s41598-021-03412-7

**Published:** 2021-12-13

**Authors:** Qingmiao Ding, Yuning Gao, Ruiyang Liu, Yaozhi Li, Lei Jin, Han Ma

**Affiliations:** 1 Aeronautical Engineering Institute, Civil Aviation University of China, Tianjin, 300300 People’s Republic of China; 2 China National Offshore Oil Corporation (CNOOC) Tianjin Pipeline Engineering Technology Co., Ltd, Tianjin, 300452 People’s Republic of China; 3 Jiangsu Shagang Group Co., Ltd., Jiangsu, 215600 People’s Republic of China

**Keywords:** Engineering, Civil engineering

## Abstract

The influence of direct current interference on the corrosion behavior of HRB400 and HRB400M steel bars in simulated concrete solution was studied using methods such as weight loss experiment, electrochemical experiment, surface technology and product analysis. The research results showed that with the increase of DC interference voltage, the corrosion rates of HRB400 and HRB400M steel bars would increase. The corrosion resistance of HRB400M steel bars was better than HRB400 steel bars under the experimental conditions. In addition, direct current interference could cause damage to the corrosion product layer on the surface of HRB400 steel bars and HRB400M steel bars. And the corrosion form and corrosion product types of HRB400 and HRB400M steel bars would be affected by direct current interference. The main corrosion products of HRB400 steel bars included γ-FeOOH and Fe_2_O_3_ when it was not interfered by DC. When DC interference was applied, the main corrosion products included Fe_3_O_4_ and Fe_2_O_3_. The corrosion products on the surface of HRB400M steel bars were mainly Fe_3_O_4_ and Fe_2_O_3_, and the types of products increased to form Cr_2_O_3_ and MnFe_2_O_4_.

## Introduction

In recent years, the rapid development of economy and the huge scale of infrastructure construction have made concrete materials widely used in various fields such as civil, industrial, and transportation. The rapid economic development had also brought about a large number of population movements. Many cities have accelerated the construction of urban rail transit because of the large passenger volume, fast, convenient and safe characteristics of railway passenger traffic^1^. The current on the track would leak into the track bed and surrounding soil media while the subway was running. Direct current would severely corrode the steel bars in nearby concrete structures^2–9^. As the use time of the reinforced concrete structure increased, external factors such as chloride ion corrosion and concrete carbonization would also cause the bearing capacity and ductility of the reinforced concrete structure to decrease. In severe cases, the building would be damaged and caused huge losses^10–14^.

The rapid development of high-voltage transmission lines and electrified railways has made the problem of stray current corrosion more prominent^15^. Stray current corrosion was divided into DC stray current corrosion and AC stray current corrosion^16^. Metros and light rails mostly used 750 V or 1500 V DC electric traction systems and track return methods in China. It was generally believed that the external concrete resistivity of the steel bar was relatively large, which played a good protective effect on the steel bar. However, when the concrete structure was damaged by carbonization and other external damage, the internal steel bar would be directly exposed to the stray current environment, which accelerated the corrosion of the steel bar. Corrosion of the steel bar would cause degradation of the bonding performance between the steel bar and concrete, and the decrease of the mechanical properties of the steel bar, resulting in a reduction in the load-bearing capacity and deformation capacity of the structure^1,5,17,18^. The bonding performance between the concrete and the steel in the reinforced concrete component was a prerequisite to ensure the cooperation between them. Corrosion of steel bars would cause bonding performance failure, greatly reducing the structural performance^19^. Therefore, the research on steel corrosion has always been an important topic for concrete structures^20^. Zhou et al.^21^ studied the corrosion of steel reinforcement in concrete under the action of stray current in the early stage. Their research results showed that compared with natural corrosion, the mass loss of steel bars under the action of stray current was greater, and the corrosion rate of steel bars was faster. The corrosion products generated by the anode reaction of the steel bars would squeeze the concrete and caused the concrete to crack. In addition, the corrosion of steel bars caused by stray current tended to be concentrated in some local locations, such as defects in the protective layer. The steel bars in these locations would quickly rust^22^. Bertolini^23^ also found that the effect of stray current would accelerate the corrosion of steel bars in concrete, and pointed out that the corrosion of steel bars interfered with DC stray current was more serious than that interfered with AC stray current. Li et al.^24^ applied stray current to reinforced concrete specimens, and studied the mechanical properties of sea sand concrete and the corrosion characteristics of steel bars under the influence of stray current through compressive strength test, elastic modulus test and electrochemical test. It was found that the stray current would accelerate the corrosion of the steel bars in the sea sand concrete, as the applied voltage increased and the energization time increased, the compressive strength and elastic modulus values of the reinforced concrete specimens were significantly reduced. He et al.^25^ used a self-made test system that simulated the interference of stray currents to measure the electrochemical polarization curves of the samples at different rotation angles to analyze and study the influence of external stray current on the electrochemical behavior of metal corrosion. The results showed that the AC stray current depolarized the metal corrosion reaction by promoting the oxygen diffusion process, and this promotion effect was strengthened with the increase of the stray current intensity.

Due to the severe impact caused by the corrosion of steel bars, corrosion-resistant steel bars have also been used as special building materials in construction projects in recent years. They have the same mechanical properties as ordinary concrete steel bars and good corrosion resistance. At the same time, because the corrosion-resistant steel bars had high corrosion resistance, the service life of the structure was prolonged and the use cost was saved. The seawater corrosion-resistant ribbed steel bar HRB400M produced by Sha gang Group has been piloted in many projects. Because of its multi-alloy element composite corrosion-resistant design, the corrosion resistance in the marine environment could be increased by more than three times compared with ordinary HRB400 steel bars, and the service life could reach 50-100a. However, HRB400M corrosion-resistant steel bars were only used in the initial stage of some projects, and there were few researches on the corrosion performance and corrosion product performance of the corrosion-resistant steel bars in the later period of use.

In order to explore the applicability of corrosion-resistant steel bars in the complex concrete environment of airports, this paper used weightlessness experiment, electrochemical experiment, surface technology, product analysis and other methods to study the influence of DC stray current on the concrete reinforcement in the later stage of carbonization. The influence of DC stray current on the corrosion behavior of HRB400 and HRB400M steel in simulated concrete solution was obtained. By comparing the experimental data of the two materials, the specific degree of corrosion resistance was analyzed in order to provide a certain reference for the wide application of corrosion-resistant steel bars in the airports.

## Experimental method

### Weightlessness experiment

The commonly used steel bars HRB400 and the new corrosion-resistant steel bars HRB400M were selected as the experimental materials. The chemical compositions (mass fraction) of the two steel bars were shown in Tables 1 and 2. The steel bar was processed into a cylinder with a size of φ20 × 3 mm by using a wire cutting machine. A circular cross-section was selected as the working surface, and the back side was soldered with copper wire. Samples were sealed with epoxy resin, and the working surface (area of 314 mm^2^) was retained. Use 320-2000 mesh waterproof sandpaper to polish the working surface gradually. After that, they were washed with acetone, absolute ethanol and deionized water, and finally dried for later use.

**Table 1:** Chemical composition of HRB400 steel bars (wt%).

	C	Si	Mn	P	V	S	Fe
Standard	0.17–0.24	0.40–0.80	1.20–1.60	≤ 0.045	0.003–0.006	≤ 0.045	Balance

**Table 2:** Chemical composition of HRB400M steel bars (wt%).

	C	Si	Mn	Cr	V	Mo	Fe
Standard	0.01–0.03	0.40–0.60	1.40–1.60	9.5–10.50	0.03–0.10	0.9–1.20	Balance

In the experiment, simulated carbonized concrete pore liquid was used, and its chemical composition was: 1000 mL H_2_O, 0.53 g/L NaHCO_3_, 1.26 g/L Na_2_CO_3_^26,27^. A DC power supply model of MCH-3205IV was used to apply DC signals of 0 V, 2.5 V, 5 V, 7.5 V, and 10 V to the test specimens. The positive pole of the power supply was connected to the working electrode, and the negative pole was connected to a carbon rod. After 100 h of corrosion under the interference of external current, the samples were taken out and rinsed with deionized water. Then put it in the rust removal solution (including 500 mL concentrated hydrochloric acid, 500 mL distilled water and 3.5 g hexamethylenetetramine) for 10 min to remove the corrosion products on the surface of the samples. Then use deionized water and absolute ethanol to clean. The samples were fully dried (the difference between the two measurements was within 0.0002 g, it could be considered as dry), and then weighed again with an electronic balance, and the corrosion rate was calculated according to formula (1).

1 $$v = \left( {m_{0} - m_{1} } \right)/St$$

In the formula, *v* was the corrosion rate, (g·m^−2^·h^−1^). *m*_0_ was the weight of the samples before the experiment, (g). *m*_1_ was the weight of the samples after the corrosion products were removed, (g). *S* was the sample area, (m^2^). *t* was the experiment time, (h).

### Electrochemical experiment

After energizing, the sample was subjected to electrochemical test. The electrochemical test was carried out on the electrochemical workstation CHI660D, and the experiment adopted a three-electrode test system. The sample was a working electrode, the Pt electrode was an auxiliary electrode, and the reference electrode was a saturated calomel electrode. Electrochemical experiments mainly included open circuit potential (OCP) and potential dynamic polarization curve testing. The open circuit potential test and the potential dynamics polarization curve test were performed on the working electrode in sequence. Set the scan rate to 0.1 mV/s and the scan range to Eocp ± 250 mV.

### Surface analysis

The surface morphology of HRB400 and HRB400M steel bars was studied by scanning electron microscope, and the corrosion morphology of the metal matrix was observed by metallurgical microscope. X-ray diffraction (XRD) was used to study the composition of corrosion products. XRD (Cu target, working voltage 40 kV, working current 40 mA, wavelength 1.5406 A, divergence slit 1 mm, cable slit 2.5°) was used to analyze the corrosion products produced under the influence of different electrical stray currents. The scanning speed was 20°/min, and the range of the scanning diffraction angle was 10°-120°.

## Experimental results and analysis

### Surface topography

**Figure 1:**
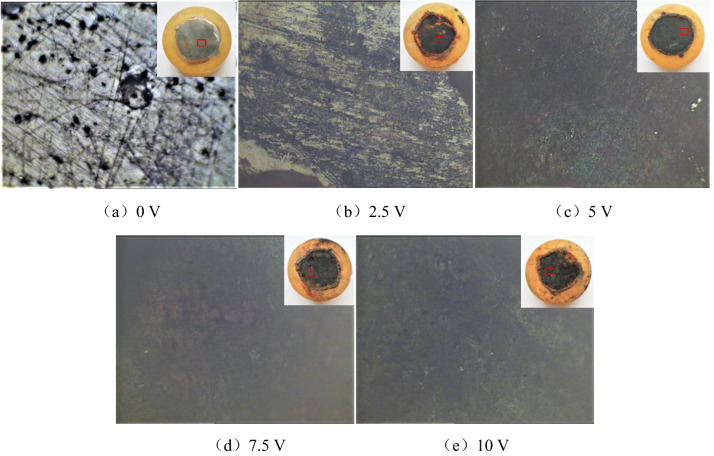
Surface corrosion morphology of HRB400 steel bars interfered with different DC voltage.

**Figure 2:**
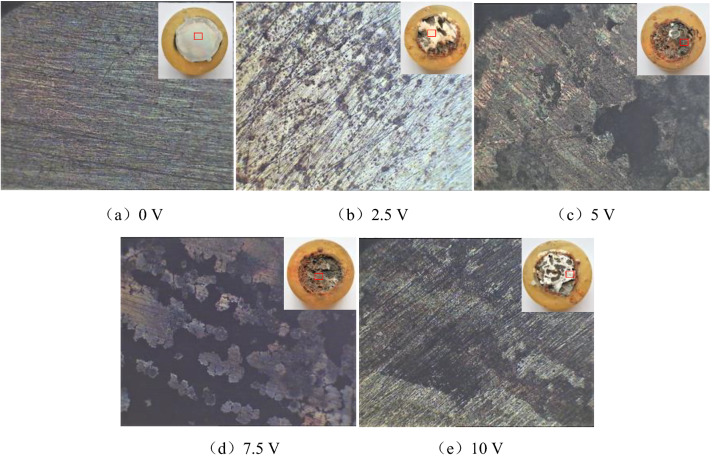
Surface corrosion morphology of HRB400M steel bars interfered with different DC voltage.

Under different degrees of direct current interference, the corrosion morphology of HRB400 and HRB400M steel bars soaked in the concrete pore liquid for 100 h were respectively shown in Figs. 1 and 2. These pictures show the surface morphology of the sample under a metallurgical microscope at 400 times magnification.

It can be seen from Fig. 1 that when there was no direct current interference (0 V) in the concrete pore liquid, pitting pits of different sizes appeared on the surface of the HRB400 steel sample, and obvious metallic luster could be seen in the remaining positions. This showed that the HRB400 steel bars would have localized corrosion after being immersed in the concrete pore liquid for 100 h. When HRB400 steel bars were interfered by DC voltage of 2.5 V, the corrosion degree of the sample surface would be deepened. The pitting pits were gradually connected, and the metallic luster area of the sample surface was obviously reduced. When HRB400 steel bars were interfered by DC voltage of 5 V, the corrosion degree of the sample was further deepened. The metallic luster of the surface disappeared completely, and the corrosion products were black and red solid oxides, mainly black oxides. When HRB400 steel bars were disturbed by DC voltage of 7.5 V, the corrosion products were also black and red solid oxides, but mainly reddish brown oxides. When HRB400 steel bars were interfered by DC voltage of 10 V, the corrosion form of the sample surface was mainly uniform corrosion. The reddish brown oxide in the corrosion product disappeared, the whole surface was black, and the degree of corrosion deepened. On the whole, the corrosion degree of HRB400 steel bars in the concrete pore fluid increased with the increase of the degree of direct current interference.

It can be seen from Fig. 2 that when there was no direct current interference in the concrete pore liquid, the surface of the HRB400M steel sample was covered by a thin layer of corrosion product film. But no obvious corrosion pits could be seen, it showed that the corrosion was not serious at this time. When HRB400M steel bars were interfered by DC voltage of 2.5 V, pitting pits of different sizes appeared on the surface of the HRB400M steel sample, and obvious metallic luster could be seen in the remaining positions. This showed that HRB400M steel bars would have localized corrosion after being immersed in concrete pore fluid for 100 h. When HRB400M steel bars were interfered by DC voltage of 5 V, the corrosion area of the sample surface increased, and the pitting pits gradually gathered together. The metallic luster on the surface almost disappeared, and the corrosion degree was more serious than that interfered with 2.5 V DC voltage. When HRB400M steel bars were interfered by 7.5 V DC voltage, the corrosion degree of the sample was further deepened, the macroscopic corrosion morphology was honeycomb, and the corrosion products were mainly black solid oxides. When HRB400M steel bars were interfered with 10 V DC voltage, the corrosion form of the sample surface was mainly local corrosion, and the corrosion product was mainly black solid oxide. Compared with 7.5 V DC interference, the corrosion area of the sample surface interfered with 10 V DC voltage was reduced, but there were more obvious corrosion pits which could be seen more intuitively. In addition, it can be seen that under the same voltage of direct current interference, the corrosion degree of HRB400M steel bars was lighter than that of HRB400 steel bars. Therefore, the corrosion resistance of HRB400M steel bars in concrete pore liquid was better than that of HRB400 steel bars.

**Figure 3:**
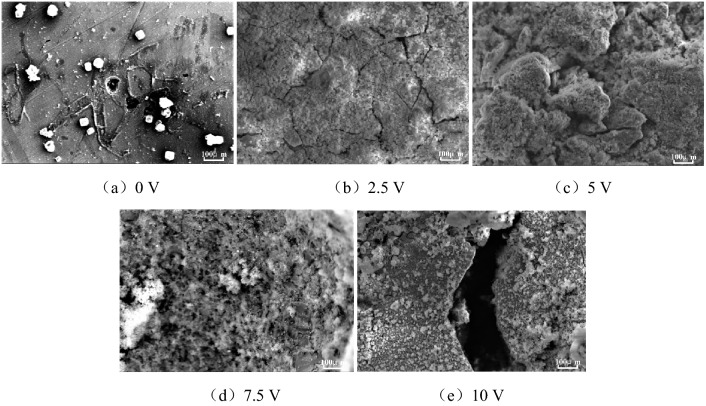
SEM images of HRB400 steel bars interfered with different DC voltage.

**Figure 4:**
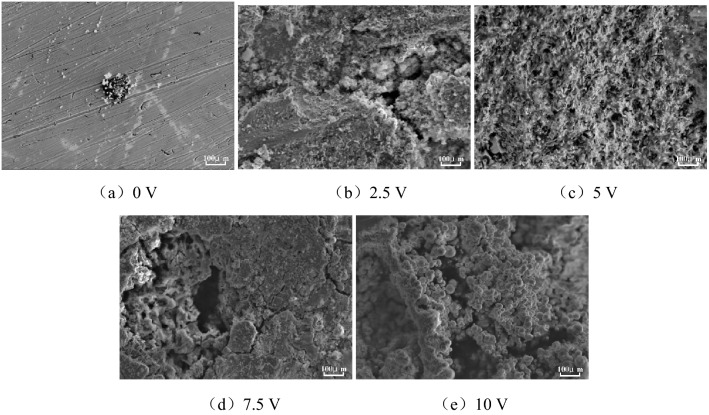
SEM images of HRB400M steel bars interfered with different DC voltage.

After being immersed in the concrete pore liquid for 100 h, the surface microscopic morphology of HRB400 and HRB400M steel bars under different voltages of direct current interference were shown in Figs. 3 and 4.

It can be seen from Fig. 3 that when there was no direct current interference in the concrete pore liquid, the corrosion products on the surface of the HRB400 steel bars were spherical, which were the typical γ-FeOOH corrosion product morphology. At the same time, corrosion pits of different sizes could be seen on the surface of the samples, which were consistent with the corrosion morphology in Fig. 1. Compared with Fig. 3a, when HRB400 steel bars were interfered with 2.5 V DC voltage, the corrosion products on the sample surface increased significantly. A stable corrosion product film formed on the sample surface, and it would protect the metal substrate. The corrosion rate of the metal substrate would decrease. When HRB400 steel bars were interfered with 5 V DC voltage, the corrosion products on the surface of the sample further increased. But the corrosion product layer was relatively loose, and the corrosion products were typical Fe_3_O_4_ morphology. When HRB400 steel bars were interfered with 7.5 V DC voltage, the corrosion products were loose and porous, and it showed a honeycomb shape. Therefore, the harmful ions in the solution would pass through the gap and further contacted the HRB400 steel matrix to promote corrosion. When HRB400 steel bars were interfered with 10 V DC voltage, the corrosion product film would appear cracks, even peeling off and other defects. On certain conditions, local corrosion such as crevice corrosion and galvanic corrosion occurred on the surface of the sample, which intensified the corrosion of HRB400 steel bars.

It can be seen from Fig. 4 that when there was no direct current interference in the concrete pore liquid, small corrosion pits appeared on the surface of the HRB400M steel bars, and the corrosion products were also typical γ-FeOOH corrosion morphologies. Compared with HRB400 steel bars, the corrosion pits on the surface of HRB400M steel bars were shallower, the quantity was less, and corrosion products were fewer. When HRB400M steel bars were interfered with 2.5 V DC voltage, the corrosion products on the surface of the samples were significantly increased compared with 0 V DC voltage, and the corrosion products were typical Fe_3_O_4_ morphology. When HRB400M steel bars were interfered with 5 V DC voltage, the corrosion products on the surface of the sample further increased. The corrosion products were distributed more uniformly, the thickness was basically the same, and the corrosion product layer of honeycomb shape was loose and porous. The microscopic appearance was similar to the HRB400 steel bars which interfered with 7.5 V DC voltage. When HRB400M steel bars were interfered with 7.5 V DC voltage, the overall corrosion product film was relatively dense, but part of the corrosion product peeled off with current interference. Therefore, in the pore liquid of reinforced concrete under the interference of 7.5 V DC, HRB400M would be more prone to local corrosion. When HRB400 steel bars were interfered with 10 V DC voltage, the corrosion products on the surface of HRB400M steel bars were black spheres, which were the typical β-FeOOH corrosion morphology. In summary, DC interference had a certain effect on the corrosion products and corrosion morphology of HRB400 and HRB400M steel bars.

### Corrosion rate

**Figure 5:**
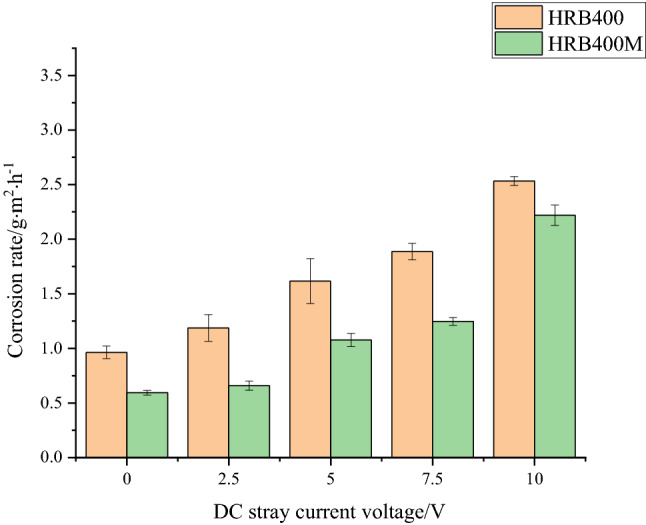
Corrosion rate of HRB400 and HRB400M steel bars with different DC voltage.

The corrosion rate of HRB400 and HRB400M steel bars after being immersed for 100 h under of different direct current interference was measured and calculated by the weightlessness method. The results were shown in Fig. 5.

It can be seen from Fig. 5 that when there was no stray current in the simulated concrete pore fluid, the corrosion rate of HRB400 steel bars after immersion for 100 h was relatively small, and the average corrosion rate was 0.9 g·m^−2^·h^−1^. With the increase of the DC interference degree in the concrete pore liquid, the corrosion rate of HRB400 steel bars increased significantly. When the DC interference voltage was 2.5 V, 5 V, 7.5 V, and 10 V, the average corrosion rate of HRB400 steel bars was 1.3 g·m^−2^·h^−1^, 1.6 g·m^−2^·h^−1^, 1.9 g.m^−2^.h^−1^ and 2.5 g·m^−2^·h^−1^. The relationship between the corrosion rate and DC interference strength of HRB400M steel bars was similar to that of HRB400 steel bars. In addition, under the same intensity of direct current interference, the average corrosion rate of HRB400M steel bars during the same immersion time was significantly lower than that of HRB400 steel bars. It indicated that the corrosion resistance of HRB400M steel bars was better than that of HRB400 steel bars.

### Electrochemical analysis

**Figure 6:**
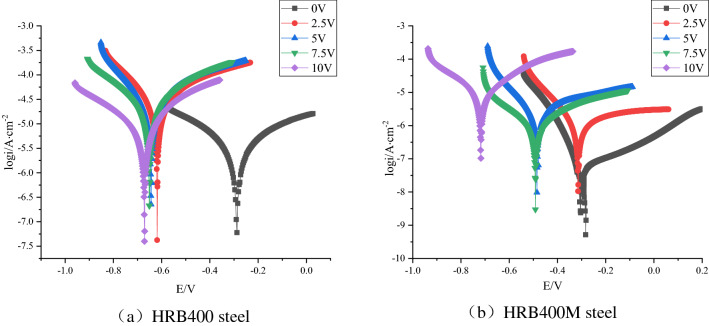
Potential polarization curves of HRB400 and HRB400M steel bars interfered with different DC voltage.

**Figure 7:**
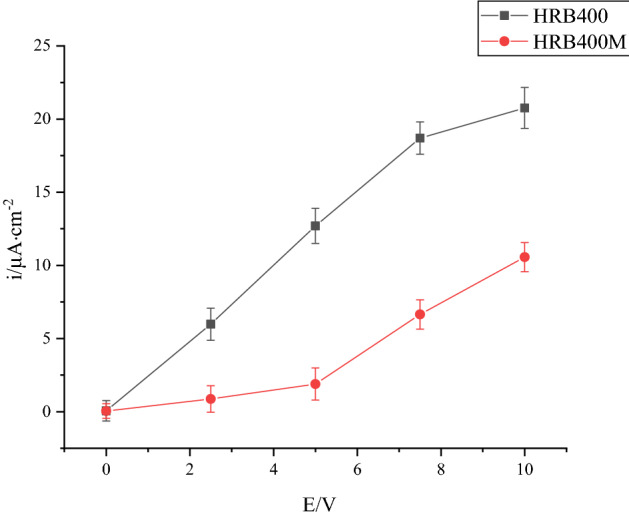
Corrosion current density of samples under the influence of different voltages on the 10th day.

The potential polarization curves of HRB400 and HRB400M steel bars interfered with different degrees of direct current in concrete pore liquid were shown in Fig. 6a,b, respectively. The polarization curve data was fitted to obtain the corrosion current density under different conditions, and the results were shown in Fig. 7.

It can be seen in Figs. 6 and 7 that the corrosion rate of HRB400 and HRB400M steel bars in the concrete pore fluid gradually increased with the increase of the degree of direct current interference. It was because Cl^−^ and O^2−^ ions in the solution underwent directional movement under the action of direct current interference, and the oxide film on the sample surface was continuously dissolved and repaired under the synergistic effect of Cl^−^ and O^2−^ plasma. The corrosion rate of HRB400 and HRB400M steel bars was faster under direct current interference. That corresponded to the chemical and electrochemical equilibrium process at the metal surface/concrete pore-liquid interface. In addition, the DC stray current would reach the surface of the metal substrate through the cracks of the corrosion product layer on the surface of the sample, and the phenomenon of "tip discharge" might appear at the defect of the product film. That would further accelerate the corrosion of HRB400 and HRB400M steel bars. At the same time, it could be seen that regardless of the voltage value in the experiment, the corrosion current density of HRB400M steel bars were lower than that of HRB400 steel bars. It showed that the corrosion resistance of HRB400M steel bars in concrete pore liquid was higher than that of HRB400 steel bars. It might be related to the low C content of HRB400M steel bars and the addition of elements such as Cr and Mn.

### Corrosion product analysis

When the experiment lasted until the second day, there was obvious black product precipitation on the surface of the test piece loaded with DC voltage.

**Figure 8:**
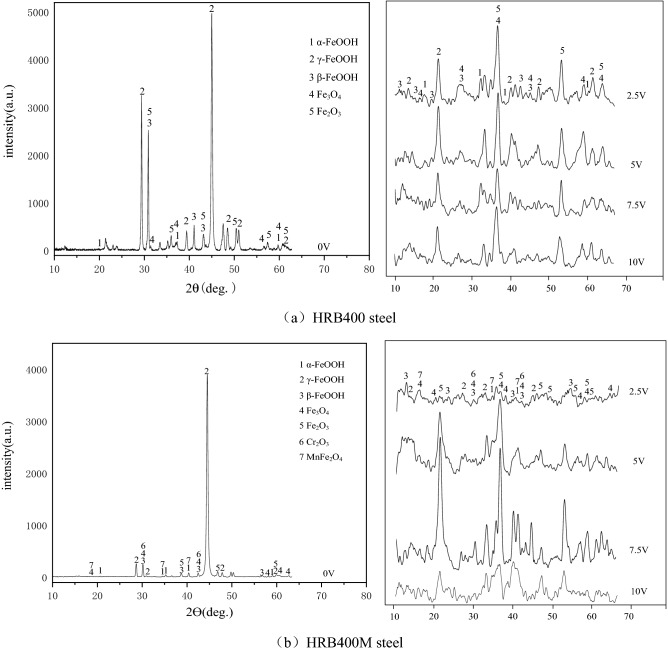
Corrosion products of HRB400 and HRB400M steel bars interfered with different DC voltage.

Interfered with different degrees of direct current, the analysis results of corrosion products of HRB400 and HRB400M steel bars immersed in the concrete pore liquid for 100 h were shown in Fig. 8. According to the results, some data with a scanning range of 10–65° were intercepted for analysis.

From the XRD test results in Fig. 8, it is found that after applying DC voltage to the two kinds of steel bars, the preferred orientation of the peaks in the XRD pattern changed. The peaks of Fe_3_O_4_ were much higher than the peaks of γ-FeOOH after the voltage applied.

It can be seen in Fig. 8a that the corrosion products on the surface of HRB400 steel bars without direct current interference were mainly γ-FeOOH and Fe_2_O_3_. When interfered with 2.5–10 V DC voltage, the characteristic peak intensity of HRB400 steel bars did not change significantly, and there were strong characteristic peaks of Fe_2_O_3_, Fe_3_O_4_ and FeOOH. It showed that the samples have been severely corroded under the DC interference of 2.5 V. When HRB400 steel bars were not interfered by direct current as shown in Fig. 8b, the corrosion products was mainly γ-FeOOH. Compared with HRB400 steel bars, the corrosion products on the surface of HRB400M steel bars increased obviously. The characteristic peak intensity of Fe_2_O_3_, Fe_3_O_4_ and FeOOH also changed in the HRB400M steel sample under the interference of 2.5–10 V DC voltage. Combining with the weight loss test of HRB400M steel bars, it was concluded that HRB400M steel bars could still maintain good corrosion resistance under the interference voltage of 2.5 V. As the voltage increased, the steel bars were corroded and black solid corrosion products appeared on the working surface. When the interference voltage was 7.5 V, the corrosion product layer was thicker than that with other interference voltages, and it resulted in higher resistance of the corrosion products, which made the metal substrate be protected to a certain extent. When the interference voltage was increased to 10 V, the corrosion product layer was broken down by the current and fell off, and that accelerated the metal corrosion. Comparing the XRD patterns of HRB400M and HRB400 materials without stray current, it was found that the FeOOH characteristic peak of HRB400 material was stronger, and Fe in the material produced more γ-FeOOH.

### Corrosion mechanism discussion

When HRB400 steel bars were corroded, Fe would rapidly oxidize to become Fe^2+^. In the simulated solution, Fe^2+^ was rapidly converted to Fe(OH)_2_ and then oxidized to FeOOH by O_2_ in the environment^28^. The main reaction process includes:

2 $${\text{Fe}} - {\text{2e}}^{ - } \to {\text{Fe}}^{{{2} + }}$$

3 $${1}/{\text{2O}}_{{2}} + {\text{2e}}^{ - } + {\text{H}}_{{2}} {\text{O}} \to {\text{2OH}}^{ - }$$

4 $${\text{Fe}}^{{{2} + }} + {\text{2OH}}^{ - } \to {\text{Fe}}\left( {{\text{OH}}} \right)_{{2}}$$

5 $${\text{Fe}}\left( {{\text{OH}}} \right)_{{2}} + {1}/{\text{2O}}_{{2}} \to {\text{2FeOOH}} + {\text{H}}_{{2}} {\text{O}}$$

**Figure 9:**
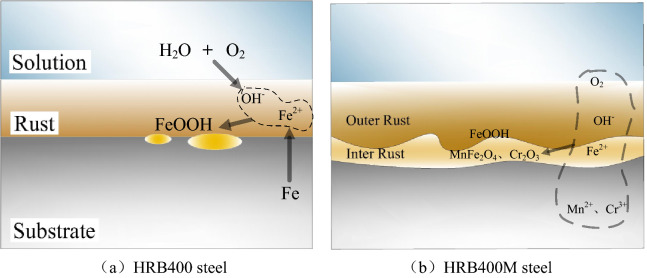
Schematic diagrams of the corrosion mechanism of HRB400 and HRB400M steel bars interfered with different DC voltage.

Combined with XRD analysis, it can be known that both HRB400 and HRB400M steel bars would produce FeOOH after corrosion. During the corrosion process of HRB400 steel bars, the interference voltage promoted the mutual transformation between the corrosion products. That made the local volume of the rust layer changed and produces forced interaction, and caused the layer of corrosion loose to be easy to fall off^29^. The schematic diagrams of the corrosion mechanism of HRB400 and HRB400M steel bars with direct current interference were shown in Fig. 9. It can be seen from Fig. 9 that the corrosion process of HRB400M steel bars was similar to that of HRB400 steel bars. However, as the corrosion progressed, metals such as Mn and Cr contained in HRB400M steel bars would be oxidized to produce Mn^2+^ and Cr^3+^. Mn^2+^ and Cr^3+^ formed MnFe_2_O_4_ and Cr_2_O_3_ in a weakly alkaline environment containing Fe^2+^, which promoted the formation of a dense oxide layer on the surface of the metal substrate and reduced the rate of formation of FeOOH. Therefore, when the interference voltage was 2.5 V, HRB400M steel bars still had better corrosion resistance. As the corrosion progressed, the FeOOH in the rust layer of the HRB400M steel bars and the Fe in the matrix would react to produce Fe_3_O_4_. The relative content of Fe_3_O_4_ increased, and its density is greater than FeOOH, and it would promote the shedding of the rust layer and accelerated the corrosion of the samples. This is because in the process of the electrochemical reaction, the applied voltage promotes the mutual transformation of the products in the electrochemical reaction. This transformation would change the local volume of the rust layer, so the rust layer would fall off and the sample would corrode faster. Therefore, when the applied voltage value increased, the corrosion resistance of HRB400M steel bars would also decrease.

## Conclusion

1. The corrosion rates of HRB400 and HRB400M steel bars in the concrete pore fluid both increased with the increase of DC interference voltage, and the corrosion resistance of HRB400M steel bars was better than that of HRB400 steel bars.
2. When there was no direct current interference, the corrosion forms of HRB400 and HRB400M steel bars in the simulated concrete pore liquid were mainly pitting corrosion. When there was direct current interference in the pore fluid, the pitting corrosion of HRB400M steel bars was intensified, and the surface was still mainly pitting corrosion, while HRB400 steel bars were mainly uniform corrosion. In addition, direct current interference would cause damage to the corrosion product layer on the surface of HRB400 steel bars and HRB400M steel bars.
3. Direct current interference would affect the types of corrosion products on the surface of HRB400 steel bars and HRB400M steel bars. When HRB400 steel bars were not interfered by direct current, the main corrosion products were γ-FeOOH and Fe_2_O_3_. The corrosion products of HRB400 steel bars interfered with direct current were mainly Fe_3_O_4_ and Fe_2_O_3_. The corrosion products on the surface of HRB400M steel samples were mainly Fe_3_O_4_ and Fe_2_O_3_, which were different from HRB400 steel bars, there would be Cr_2_O_3_ and MnFe_2_O_4_ in the corrosion products of HRB400M steel bars. Cr and Mn would promote the formation of a dense oxide layer on the sample surface to a certain extent, and reduce the formation rate of FeOOH to inhibit corrosion.

## Data Availability

The data used to support the findings of this study are included within the article.
